# Mammary Myofibroblastoma in a Male: A Case Report and Review of the Literature

**DOI:** 10.7759/cureus.35335

**Published:** 2023-02-22

**Authors:** Jumana A Fatani, Ohood Alotaibi, Mhmmd Jacob, Khalil Terro, Khalid Alhajri

**Affiliations:** 1 General Surgery, Specialized Medical Center, Riyadh, SAU; 2 General Surgery/Breast and Endocrine, Prince Sultan Military Medical City, Riyadh, SAU

**Keywords:** tumor, stromal, male, breast, myofibroblastoma

## Abstract

Mammary myofibroblastoma is a rare benign tumor. It is mainly seen in older men and postmenopausal women. These tumors can be presented with a palpable mass or can be discovered incidentally on routine screening. A 76-year-old male presented with a palpable breast mass that was increasing in size. The patient underwent wide local excision with no postoperative complications. The pathology finding was consistent with myofibroblastoma. Myofibroblastoma is a rare tumor and should be considered one of the differential diagnoses in breast lumps.

## Introduction

Myofibroblastoma is a benign stromal tumor that has been identified in mammary and extramammary regions [1]. Mammary myofibroblastoma (MFB) is rare, with similar rates of occurrence among males and females and greater incidences in older versus younger men and postmenopausal versus premenopausal women [2,3]. These tumors can be symptomatic or asymptomatic and are often discovered incidentally [1]. To date, fewer than 90 cases of MFB have been reported [2]. We present a case of a 76-year-old male who presented with a palpable breast mass. Biopsy identified the mass as an MFB, which was then surgically excised.

## Case presentation

A 76-year-old male with known hypertension and unremarkable surgical history presented with a two-year history of a painless right breast mass that was increasing in size and no other concerns. On examination, a retroareolar right breast mass was observed, measuring about 5 cm, with no palpable axillary or supraclavicular lymph nodes. No skin changes, nipple retraction, or discharge was observed.

A bilateral breast ultrasound showed a 54 x 41 x 53 mm retroareolar soft tissue lesion on the right breast. The mass was mainly iso- and hyperechoic with scattered hypoechoic masses showing likely necrotic areas. There were no obvious calcifications. Mild internal vascularity was noticed. His left breast had no obvious soft tissue lesions and no pathological axillary lymph nodes. Differential diagnoses at this stage included breast cancer and liposarcoma.

**Figure 1:**
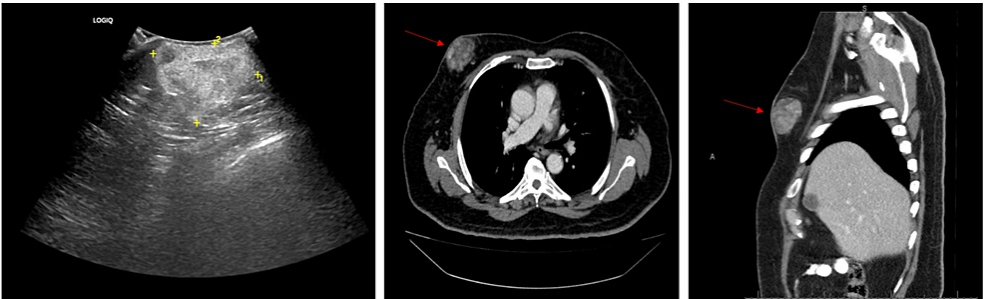
Diagnostic images (A) Ultrasound of right breast showing soft tissue lesion, (B) Axial computer tomography of chest showing soft tissue lesion (red arrow); (C) Coronal computer tomography of chest showing soft tissue lesion (red arrow).

Computer tomography of the chest with intravenous contrast revealed a 53 x 40 x 55 mm heterogeneous soft tissue lesion of the right breast in the retroareolar region, with internal fatty areas and moderate heterogeneous enhancement on the postcontrast study (Figure 1) that was worrisome for malignancy. A few small and likely insignificant right axillary lymph nodes were noted. No significant mediastinal or hilar masses were observed.

**Figure 2:**
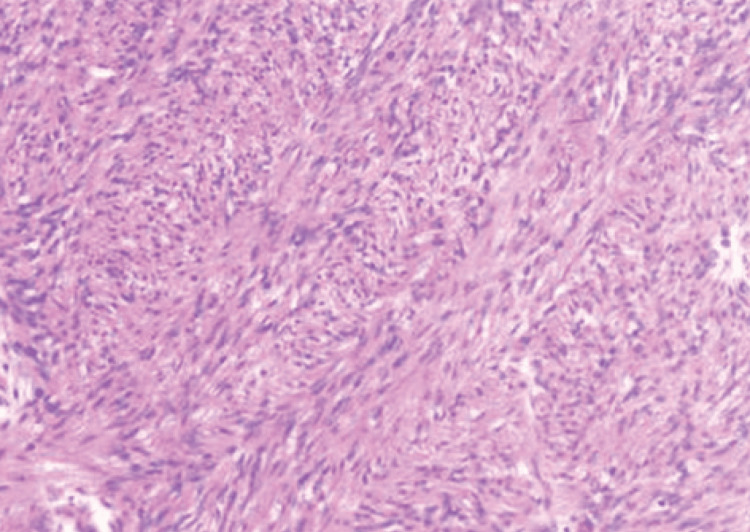
Histology image (low‑power view) showing a tumor composed of fascicles of benign spindle cells Image obtained from reference [1] and included here for demonstration purposes.

Core needle biopsies were taken from the right breast mass and right axillary lymph node. Results were consistent with myofibroblastoma, and the right axillary lymph node indicated reactive lymphoid tissue that was negative for malignancy. The patient underwent wide local excision of the right breast mass with margins. On gross examination, the rounded mass measured 55 x 50 x 35 mm. A cut section revealed a grayish-yellow, rubbery, cut surface. Histopathology showed a benign spindle-cell tumor of the mammary stroma composed of fibroblasts and myofibroblasts and consistent with short fascicles of bland spindled cells, dense hyalinized collagen bundles, and focal areas of fatty tissue. No nuclear atypia, mitosis, or necrosis was identified. Many mast cells and focal myoxid areas were observed. The demonstration of fascicles of benign spindle cells is shown in (Figure 2). This image is obtained from a previous study and included here for demonstration purposes [1]. The tumor size was 5.5 cm on its longest axis. All margins were negative for malignancy. The diagnosis was consistent with myofibroblastoma. Immunohistochemical stains were positive for desmin protein and negative for CD34, S100, and PAN-CK, thus confirming the diagnosis of myofibroblastoma.

## Discussion

Myofibroblastoma is a rare, benign, stromal tumor of the mesenchyma with myofibroblastic differentiation [2,3]. It has been reported in mammary and extramammary regions [2], including the head, neck, soft tissue, skin, lymph node, axilla, parotid gland, and groin [4-7], with similar histology and immune‑phenotyping [1]. MFB was first described in 1987 by Wagortz et al., who reported 16 cases (11 in men) [8]. Extramammary myofibroblastoma was first described in 2001 by McMenamin and Fletcher [9]. Although initially described and thought to be more prevalent in males, MFB affects females with a similar incidence, probably due to increased mammographic screening [1,10,11]. Tumors may be detected earlier in women due to routine mammary screening, whereas in men MFB usually presents as a painless palpable mass [1]. It is mainly seen in older men and postmenopausal women aged 40-87 years [3]. 

A review of the literature revealed 43 cases of MFB (25 men, 17 women, and one transgender patient aged 35-96 years). Among them, 24 were incidental findings on routine mammography screening [2] or other screenings for unrelated symptoms [1,12], and 19 involved a palpable mass or other symptoms [3]. Differential diagnoses for breast mass in males include gynecomastia, infection, lipoma, granular cell tumor, metastatic disease, and schwannoma [13]. See Table 1 for a summary of the literature review.

**Table 1:** Literature review

Article	Age	Gender	Presentation	Management
Khatib et al., 2018 [2]	55	Female	Breast lump detected on routine mammography (incidental)	Lumpectomy + intraoperative frozen section
Scardina et al., 2021 [3]	56	Male	Lump in the mammary region	Complete excision with the overlaying skin, preserving the nipple
Wickre et al., 2021 [4]	Males: 50-96 Females: 52-89	Males: 12 Females: 8	Lump: 9 Routine screening: 11	Surgical excision: 8 Imaging follow-up: 3 Lost to follow-up: 9
Strait et al., 2021 [12]	70	Male	Persistent cough (incidental)	Surgical excision
Bağlan et al., 2021 [14]	62	Male	Chronic obstructive pulmonary disease (incidental)	Surgical resection
Fügen et al., 2016 [15]	35	Female	Breast lump	Excisional biopsy
Jung et al., 2020 [16]	52 61	Female Female	History of cervical cancer lump discovered on routine imaging (incidental) History of colon cancer lump discovered on computer tomography scan for preoperative evaluation (incidental)	Wide local excision Surgical excision
Venturelli et al., 2020 [1]	65 76	Male Male	Palpable mass in the breast Severe cough for a long period lump discovered on chest computer tomography scan (incidental)	Radical left mastectomy with axillary dissection Radical left mastectomy and the removal of the sentinel lymph node.
O'Bryan et al., 2018 [17]	76	Transgender (male to female)	Incidental	Surgery
Boudaouara et al., 2017 [18]	43	Female	Breast nodule diagnosed radiologically (incidental)	Surgical excision biopsy
Akrami et al., 2019 [13]	65	Male	Breast mass	Modified radical mastectomy
Jing et al., 2017 [19]	42	Female	Painless lump in the mammary region	Surgical excision
Allahverdi and Allahverdi, 2017 [5]	61	Male	Right breast mass	Excision
Ross et al., 2019 [11]	36-40	Female	Sensation of right axillary fullness (incidental)	Excisional biopsy
Shanmugasiva et al., 2018 [6]	80	Male	Right breast enlargement	Wide local excision
Yilmaz et al., 2018 [20]	53	Male	Palpable, rapidly growing mass on left breast that is	Nipple-sparing mastectomy
Comer et al., 2017 [21]	73	Male	Soft tissue mass of the left retroareolar chest wall on computer tomography assessment for multiple genitourinary malignancies (incidental)	Wide local excision
Fakim et al., 2019 [7]	52	Female	Computer tomography for recurrent sore throat (incidental)	Ultrasound-guided Vacora breast biopsy system
Viswanathan et al., 2017 [22]	74	Male	Bilateral breast masses	Clinical observation
Shintaku et al., 2017 [23]	56	Female	Painless induration in the right breast	Excision
Gambre et al., 2019 [24]	93	Male	History renal cell carcinoma, computer tomography follow‐up evaluation (incidental)	Not excised
Rochlis and Germaine, 2017 [25]	50	Male	Hemoptysis (incidental)	Surgical resection

Myofibroblastoma has no genetic tendency, and most cases are sporadic. No association between MFB and ethnicity [1,3], gender, medical condition, or medication [3] has been established. A few cases describe gynecomastia, chest wall trauma, surgical site scar incision, and breast cancer radiation as possible contributing factors [2,13]. These tumors also can occur after hormone stimulation and have been linked to gynecomastia [2,10,13,21]. In one case, a transgender patient developed an estrogen receptor positive MFB after receiving estrogen therapy for 13 months [17]. MFB also may be misdiagnosed as other benign or malignant breast diseases [1], particularly because clinical and radiological evidence may be suggestive of fibroadenoma [10,11]. Clinical presentation is characterized by a solid, solitary, slow-growing, mobile, well-circumscribed, and painless palpable mass that is firm to solid [3,10,13,18]. Rapid growth thus may raise suspicion for phyllodes tumor [10]. MFB typically ranges from 1-4 cm but can reach 16 cm in size [18,25]. On gross examination, MFB is a solid, defined, encapsulated tumor beside foci of mucoid and lipomatous changes. Cystic changes, hemorrhage, and necrosis are rare [16]. 

Radiological evidence of MFB is nonspecific. Ultrasonography is usually the first line in diagnosing any breast mass [13] and distinguishing between cystic and solid masses [5]. On ultrasonography, MFB is well demarcated and shows a variable and mixed‑echo pattern often classified as a benign lesion [2,3].

Mammography usually shows a heterogeneous, encapsulated, well‑defined border with no microcalcifications [2,3], for which the differential diagnoses include leiomyoma, hematoma, abscess, neurofibroma, lymphangioma, and cystic fibroadenoma. The differentials for malignant lesions include sarcoma, lymphoma, malignant fibrous histiocytoma, phyllodes tumor, and breast cancer [5]. 

As imaging findings are nonspecific, diagnosis is usually confirmed via biopsy to evaluate histopathology and immunohistochemistry [2,5]. Needle biopsy is associated with higher likelihood of misdiagnosis or inconclusive results, but excisional biopsy can be diagnostic and therapeutic [5]. Features of MFB on histopathology include spindle cells in fascicles with collagen bundles, well-demarcated borders, low mitotic activity, and positive CD34 [2,3]. Many additional variants of MFB have been identified since the first reported cases, including collagenous, fibrous, cellular, lipomatous, infiltrative, epithelioid, myxoid, palisaded, and decidua-like variants [2,6,16]. Immunohistochemistry thus is essential to confirm the diagnosis [2]. Tumor markers that manifest myofibroblastic differentiation include α‑SMA, desmin, vimentin, and CD34 [2]. 

MFB can be diagnosed before surgery via fine-needle aspiration cytology [18]. The current treatment is local surgical excision [3,10]. Solid, well-capsulated tumors have good cleavage plane, which makes surgical excision easier [3]. MFB is unlikely to relapse if the resected margins are free [3]. No cases of malignant transformation or recurrence have been reported [2,3].

## Conclusions

MFB is a rare benign tumor that usually presents as a palpable mass in older men and women. We describe a case involving a 76-year-old male who presented with a palpable breast mass that was surgically removed with no postoperative complication. This case highlights the importance of considering this rare tumor type as a differential diagnosis in breast lumps, especially in those who are not screened regularly for breast cancer. Any breast masses should be treated as suspicious to rule out malignancy and improve patient outcomes.
